# Bone Marrow-Derived Mesenchymal Stem Cells Differentially Affect Glioblastoma Cell Proliferation, Migration, and Invasion: A 2D-DIGE Proteomic Analysis

**DOI:** 10.1155/2021/4952876

**Published:** 2021-02-11

**Authors:** Shenjie Li, Wei Xiang, Junjie Tian, Haorun Wang, Shuiwang Hu, Ke Wang, Ligang Chen, Changren Huang, Jie Zhou

**Affiliations:** ^1^Department of Neurosurgery, The Affiliated Hospital of Southwest Medical University, Southwest Medical University, Luzhou, Sichuan 646000, China; ^2^Neurosurgery Clinical Research Center of Sichuan Province, Luzhou 646000, China; ^3^Academician (Expert) Workstation of Sichuan Province, Luzhou 646000, China; ^4^Neurological Diseases and Brain Function Laboratory, Luzhou 646000, China; ^5^Guangdong Provincial Key Laboratory of Proteomics, Department of Pathophysiology, School of Basic Medical Sciences, Southern Medical University, Guangzhou 510515, China

## Abstract

Bone marrow-derived mesenchymal stem cells (BM-MSCs) display high tumor tropism and cause indirect effects through the cytokines they secrete. However, the effects of BM-MSCs on the biological behaviors of glioblastoma multiforme remain unclear. In this study, the conditioned medium from BM-MSCs significantly inhibited the proliferation of C6 cells (*P* < 0.05) but promoted their migration and invasion (*P* < 0.05). Two-dimensional fluorescence difference gel electrophoresis (2D-DIGE) proteomic analysis revealed 17 proteins differentially expressed in C6 cells exposed to the BM-MSC-conditioned medium including five upregulated proteins and 12 downregulated proteins. Among these, six differentially expressed proteins (Calr, Set, Oat, Npm1, Ddah1, and Tardbp) were closely related to cell proliferation and differentiation, and nine proteins (Pdia6, Sphk1, Anxa4, Vim, Tuba1c, Actr1b, Actn4, Rap2c, and Tpm2) were associated with motility and the cytoskeleton, which may modulate the invasion and migration of tumor cells. Above all, by identifying the differentially expressed proteins using proteomics and bioinformatics analysis, BM-MSCs could be genetically modified to specifically express tumor-suppressive factors when BM-MSCs are to be used as tumor-selective targeting carriers in the future.

## 1. Background

Glioma is the most common primary intracranial tumor, and glioblastoma multiforme is the most malignant subtype, with a median overall survival time of 14.6 months [1]. Despite technological advances in surgery, chemotherapy, and radiotherapy in recent years, glioma patients' prognoses remain unsatisfactory [2]. Recent studies have indicated that stem cell-assisted gene therapy can suppress cancer cells that are resistant to conventional treatments. It has the potential to become a promising treatment strategy for malignant gliomas in preclinical animal models [3].

Bone marrow-derived mesenchymal stem cells (BM-MSCs) are nonhematopoietic stem cells derived from the bone marrow microenvironment. They can self-renew, form colonies, and differentiate into multiple mesodermal cell types. Compared with embryonic or neural stem cells (NSCs), BM-MSCs are increasingly being developed for potential clinical use because they are easily obtained from patients, easily cultivated and isolated, and readily engineered to deliver therapeutic agents [4]. Furthermore, previous studies have reported that BM-MSCs can cross the blood-brain barrier and display tumor-tropic properties in glioma-related models [5]. Therefore, BM-MSCs have emerged as an attractive carrier for delivering therapeutic genes to tumors and diseased tissues [6, 7].

BM-MSCs can cause a direct effect through intercellular signaling via physical contact with tumor cells and an indirect effect through the secretion of cytokines. However, the role of BM-MSCs in the biological behaviors of various cancers remains controversial [8–10]. In this study, we aimed to explain the influence of BM-MSCs on rat C6 cells *in vitro* and *in vivo* to explore the role of BM-MSCs in the tumor microenvironment. Moreover, we identified differentially expressed proteins between C6 and C6 treated with BM-MSC-conditioned medium using two-dimensional fluorescence difference gel electrophoresis (2D-DIGE) in combination with matrix-assisted laser desorption ionization time-of-flight mass spectrometry (MALDI-TOF/TOF MS). The potential molecular markers contributing to BM-MSC functions were also explored.

## 2. Methods

### 2.1. Experimental Animals

Sprague-Dawley (SD) rats (male, 3 weeks old) and nude mice (female, 10 days old) were provided by the Laboratory Animal Centre of Southwest Medical University (license number: SCXK (CHUAN) 2013–17). The institutional ethical committee of the affiliated hospital of Southwest Medical University approved all experimental animal studies. The tumor burden did not exceed the recommended dimensions according to the University of Pennsylvania IACUC guidelines, and the animals were anesthetized and sacrificed using acceptable methods.

### 2.2. Cell Cultures

Rat C6 glioma cells were cultivated in low-glucose Dulbecco's modified Eagle's medium (DMEM) (Gibco, Life Technologies, Grand Island, NY, USA) supplemented with 1% streptomycin-penicillin solution (Gibco), 10% fetal bovine serum (FBS; Gibco), and 2 mM GlutaMAX (Gibco) at 5% СО_2_ and 37°C in a humidified atmosphere.

### 2.3. BM-MSC Isolation, Culture, and Characterization

BM-MSCs were isolated as described previously [11]. Briefly, the bone marrow of euthanized 3-week-old male SD rats was obtained by flushing the marrow cavity of their femurs with low-glucose DMEM (Gibco) supplemented with 10% FBS (Gibco) and 1% streptomycin-penicillin solution (Gibco). After centrifugation, the cells were resuspended and cultured at 5% СО_2_ and 37°C in a humidified atmosphere. After 2 days, the nonadherent cells were removed, and the medium was changed every 2–3 days. BM-MSCs at the third passage were characterized using flow cytometry analysis, and BM-MSCs from the third and fourth passages were used for the following experiments. The presence of the surface markers of BM-MSCs (CD29, CD34, CD45, and CD90) was verified using flow cytometry with the appropriate primary labeled antibodies (Abcam, Cambridge, UK). The differentiation of osteogenic cells and fat cells for BM-MSCs was evaluated by Alizarin red staining and oil red staining, respectively.

### 2.4. Preparation of the Conditioned Medium

The BM-MSCs were cultured in low-glucose DMEM (Gibco) supplemented with 1% streptomycin-penicillin solution (Gibco), 2 mM GlutaMAX (Gibco), and 10% FBS (Gibco) at 5% СО_2_ and 37°C in a humidified atmosphere until 70–80% confluence was achieved. Then, the growth medium was removed, and the BM-MSCs were washed with phosphate-buffered saline (PBS, Gibco), and a fresh portion of the growth medium was added. After 24 h of incubation, the medium was transferred to a 15 ml tube and then centrifuged (3,000 rpm/10 min). Subsequently, the BM-MSC-conditioned medium was transferred into a new tube and used in the subsequent experiments. The BM-MSC-conditioned medium can be frozen and stored at -80°C.

### 2.5. Treatment of C6 Cells with the Conditioned Medium Derived from BM-MSCs

The C6 cells were treated with a mixture of low-glucose DMEM and BM-MSC-conditioned medium (5 : 5) containing 10% FBS at 5% СО_2_ and 37°C for 72 h (C6-A_1_), and the culture medium was replaced every 24 h during this time. Then, a portion of the C6-A_1_ cells was trypsinized at 70–80% confluence (0.1% ethylenediaminetetraacetic acid (EDTA) with 0.25% trypsin, Gibco) and subcultured with low-glucose DMEM containing 10% FBS to the next passage (C6-A_1-1_) at 5% СО_2_ and 37°C. C6-A_1-1_ cells were trypsinized until 70–80% confluence again and subcultured with low-glucose DMEM containing 10% FBS to the third passage (C6-A_1-3_). Meanwhile, a portion of C6-A_1_ was trypsinized and subcultured with a mixture of low-glucose DMEM and BM-MSC-conditioned medium (5 : 5) containing 10% FBS to the next passage (C6-A_2_) sequentially and subcultured with low-glucose DMEM containing 10% FBS to the third passage (C6-A_2-3_), according to the above-mentioned method. Furthermore, C6-A_3-3_ cells were generated following the above-mentioned method. C6 cells were cultured in the standard medium as a control group (see a diagrammatic drawing, Figure S2).

### 2.6. Cell Proliferation Assay

C6 control, C6-A_1-3_, C6-_A2-3_, and C6-_A3-3_ cells were plated in triplicate into 96-well culture plates (2,000 cells/well) in growth medium and cultured at 37°C and 5% CO_2_. Next, 10 *μ*l/well of reagent (cell counting kit-8 (CCK-8); CK04, Dojindo, Japan) was added at 24, 48, 72, and 96 h after plating. Optical densities at a wavelength of 450 nm (OD450) were measured after 3 h of CCK-8 incubation at 37°C and 5% CO_2_, using a microplate reader (Wellscan MK3, Thermo LabSystems, Finland). The assays were independently repeated at least three times.

### 2.7. Scratch Migration Assay

C6 control, C6-A_1-3_, C6-_A2-3_, and C6-_A3-3_ cells (1 × 10^6^ cells/well) were seeded until confluence in 24-well plates. A straight scratch was gently made using a 200 *μ*l pipette tip, and the cells were cultured in serum-free medium. Images were captured 24 h after scratch generation using an inverted phase-contrast microscope (Leica, Germany), and the area of the wound was quantified using ImageJ software. The assays were independently repeated at least three times.

### 2.8. Matrigel Invasion Assay

Transwell inserts (8 *μ*m pore size, Corning, New York, NY, USA) were precoated with Matrigel (BD Biosciences, Franklin Lakes, NJ, USA) to form a matrix barrier. C6 control, C6-A_1-3_, C6-_A2-3_, and C6-_A3-3_ cells (2 × 10^4^ cells/200 *μ*l) were suspended in serum-free medium and added to the upper chamber. Medium with 10% FBS was added to the lower chamber. After 48 h of incubation at 37°C in a 5% CO_2_ atmosphere, the cells in the upper membrane were removed. Cells that had invaded through the membrane were fixed with 4% phosphate-buffered paraformaldehyde and stained with 0.1% crystal violet (Solarbio Life Sciences, Beijing, China). Cells were counted and photographed using an inverted phase-contrast microscope (Leica, Germany). Quantification of cell invasion was presented as the average calculation of stained cells in five random fields of each filter. The assays were independently repeated at least three times.

### 2.9. *In Vivo* Tumor Model

C6 control, C6-A_1-3_, C6-_A2-3_, and C6-_A3-3_ cells were diluted in PBS and subcutaneously injected into the right armpit of 10-day-old nude mice. Every mouse was injected with 2 × 10^6^ cells/0.2 ml. Finally, six mice in each group were euthanized after 14 days (tumor volume = 1/2; long tumor diameter × short tumor diameter^2^). A piece of the tumor tissue from each animal was fixed in 4% paraformaldehyde for pathological examination.

### 2.10. Immunohistochemistry (IHC)

Paraffin sections (4 *μ*m) were deparaffinized in 100% xylene and rehydrated in a descending ethanol series and water according to standard protocols. Heat-induced antigen retrieval was performed in 10 mM citrate buffer for 2 min at 100°C. Endogenous peroxidase activity and nonspecific antigens were blocked with a peroxidase-blocking reagent containing 3% hydrogen peroxide and serum, and then, rat anti-nestin (7A3; Abcam) and rabbit anti-MMP-9 (ab38898, Abcam) were incubated overnight at 4°C. After washing, the sections were incubated with biotin-labeled goat anti-rat antibody and goat anti-rabbit antibody for 30 min at room temperature and subsequently incubated with streptavidin-conjugated horseradish peroxidase (HRP) (S911, Thermo Fisher). The peroxidase reaction was developed using 3,3-diaminobenzidine (DAB) chromogen solution in DAB buffer substrate. Sections were visualized with DAB and counterstained with hematoxylin, mounted in neutral gum, and analyzed using a bright field microscope. The intensity of staining was scored semiquantitatively as negative (score 0), weak (score 1), moderate (score 2), or strong (score 3), as described previously [12]. For statistical evaluation, scores of 0 and 1 were considered negative (-), while scores of 2 or 3 were positive (+).

### 2.11. Protein Determination, 2D-DIGE, and Protein Identification

The total proteins were extracted from C6 control and C6-A_3-3_ cells with 500 ml of lysis buffer and then incubated on ice for 30 min. Suspensions were sonicated five times using a U200S sonicator (IKA Labortechnik, Germany) and then centrifuged for 30 min (12,000 g). The suspension proteins were then precipitated using a 2-D Clean-Up Kit (GE Healthcare) and resuspended in lysis buffer. The protein content of C6 and C6-A_3-3_ was determined using a 2-D Quant Kit (GE Healthcare). All samples were stored at -80°C before electrophoresis.

For DIGE, proteins (50 *μ*g) were minimally labeled with CyDye DIGE fluors (400 pmol Cy3 or Cy5 protein-labeling dye, GE Healthcare). Cy2 was used as the internal standard, and Cy3 or Cy5 were used as the internal standard. Each labeled sample was mixed with rehydration buffer (GE Healthcare) and applied to a 24 cm immobilized pH gradient gel strip (pH 3–10 NL) for separation in the first dimension. First-dimension isoelectric focusing was performed at 20°C in IPGphor III (GE Healthcare). After that, strips were equilibrated and loaded onto a polyacrylamide gel (12%) and then subjected to an electric field in DALTsix (GE Healthcare) at 15°C for 12 h. After 2-DE electrophoresis, gels were scanned using a Typhoon 9400 imager (GE Healthcare) and analyzed using DeCyder 2D software V6.5 (GE Healthcare). The differential protein spots were selected (filtering conditions: at least 50% change in the ratios between groups followed by a *t* test with *P* < 0.05). The matched protein spots were detected automatically with an Ettan Spot Picker (GE Healthcare).

For protein identification, the selected protein spots were destained, dehydrated, dried, and digested in order. The digested peptide mixtures from each gel spot were extracted and dried. Then, 0.5 *μ*l of matrix solution was added to the dried samples, and the samples were air-dried. Samples were then analyzed using an ABI 4800 Proteomics Analyzer MALDI-TOF/TOF MS (Applied Biosystems). The mass spectrometry (MS) and MS/MS spectra were combined and used for database searches using MASCOT software (Matrix Science, version 2.1). GPS Explorer™ software version 3.6.2 (Applied Biosystems) was used to create and search files with MASCOT. Protein identification was performed using the MASCOT search engine against Swiss-Prot nonredundant sequence databases selected for rat taxonomy. For the MASCOT search, the search parameters were as follows: peptide mass tolerance, ±50 ppm; fragment mass tolerance, ±0.25 Da; peptide charge, +1; carbamidomethylation of cysteine as fixed modification and oxidation of methionine as variable modification; trypsin digestion with maximum one missed cleavage; total sequences for Swiss-Prot database, 8128 sequences; and taxonomy species, Rattus norvegicus. For the MASCOT database search of the PMF MS spectrum, protein hits with scores greater than 26 were considered significant (*P* < 0.05) (ion score is −10∗log(*P*), where *P* is the probability that the observed match is a random event). In case no proteins could be identified from the first spectrum, additional database searches of the automatically generated MS/MS spectra were performed (analog: *P* < 0.05). The validation of the function and distribution of the identified differential proteins, were analyzed by using PANTHER13.1 (http://pantherdb.org/).

### 2.12. Western Blot Analysis

Western blot analysis was performed as described previously [13, 14], with primary antibodies including the following: rabbit anti-PDIA6 (1 : 1,500; Abcam, Cambridge, MA, USA), anti-beta-centractin (1 : 500; Santa Cruz Biotechnology, Inc., Santa Cruz, CA, USA), and anti-*β*-actin antibody (1 : 2,000; Cell Signaling Technology, Beverly, MA, USA). HRP-conjugated secondary antibody (Santa Cruz Biotechnology) was used as the secondary antibody. The protein signals were detected using SuperSignal (R) West Femto Maximum Sensitivity Substrate (Thermo Scientific Pierce). The grayscale value quantified using ImageJ software was used to calculate relative protein expression. The assays were independently repeated at least three times.

### 2.13. Statistical Analysis

In this study, all experiments were repeated at least three times, and all data were expressed as the mean ± standard deviation. The Hartley test analyzed the homogeneity of variance. Data were analyzed with the least significant difference *t* (LSD-t) test when the variance was homogeneous or Dunnett's T3 when the variance was not homogeneous. Statistical analyses were performed using the SPSS 20.0 software package (SPSS, Chicago, USA). *P* < 0.05 was considered statistically significant.

## 3. Results

### 3.1. Analysis of Rat BM-MSC Antigen Expression and the Osteogenesis and Differentiation of Fat Cells from BM-MSCs

**Figure 1:**
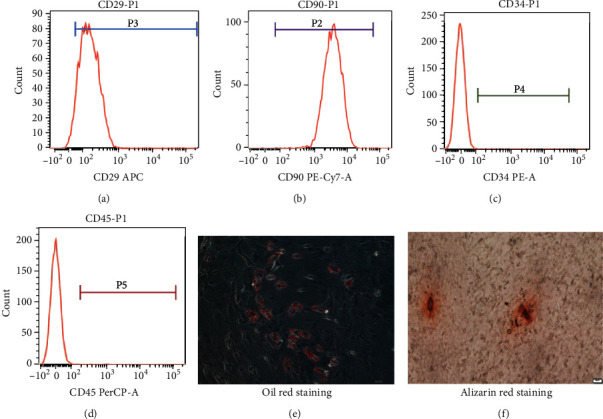
The identification of BM-MSC. BM-MSC-related specific markers, including CD29 (a) and CD90 (b), were expressed in BM-MSCs, whereas hematopoietic stem cell markers, including CD34 (c) and CD45 (d), were not identified by flow cytometry. The culture demonstrated numerous intracellular lipid droplets by oil red staining and some osteoblasts by Alizarin red staining in the BM-MSCs (e, f).

Approximately 95.32% and 99.97% of the rat BM-MSCs were positive for the expression of the typical mesenchymal surface markers CD29 and CD90, respectively, but negative for hematopoietic surface markers CD34 and CD45 (Figure 1(a)). The oil red staining of BM-MSCs indicated numerous lipid droplets. Some osteoblasts were observed following Alizarin red staining (Figure 1(b)). These results are similar to those of a previous study on the phenotype of rat BM-MSCs [15].

### 3.2. Inhibition of C6 Cell Proliferation and Promotion of Migration and Invasion by *In Vitro* BM-MSC-Conditioned Medium Treatment

**Figure 2:**
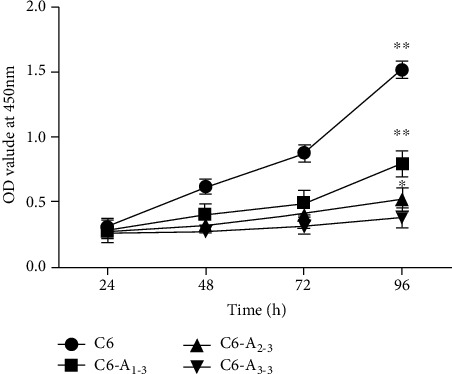
The proliferation of C6 cells treated with BM-MSC-conditioned medium. The proliferation of glioma cells showed a decreased tendency with the BM-MSC-conditioned medium treatment time extension by CCK-8 assays. Absorbance was read at 450 nm. ^∗^*P* < 0.05, ^∗∗^*P* < 0.01; error bars indicate ±SEM.

CCK-8 data showed that the proliferation of C6-A_1-3_, C6-A_2-3_, and C6-A_3-3_ cells decreased significantly compared to that of the C_6_ control group after 24 h (*P* < 0.05). Moreover, glioma cell proliferation showed a decreasing tendency with prolonged BM-MSC-conditioned medium treatment time (Figure 2).

**Figure 3:**
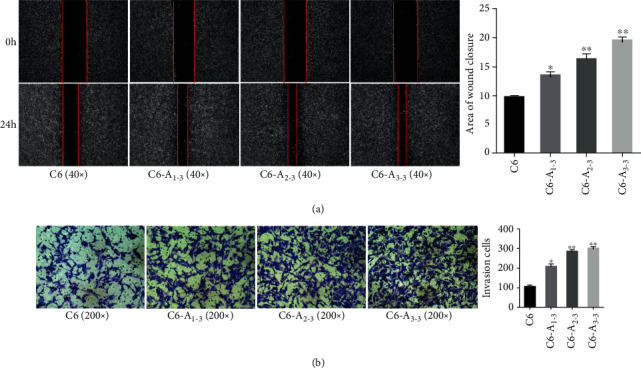
Migration and invasion of C6 cells treated with BM-MSC-conditioned medium. The migration and invasion of C6 cells showed an increased tendency with the BM-MSC-conditioned medium treatment time extension in (a) scratch migration assays for 24 h (40x magnification) and (b) transwell assay (200x magnification), respectively, *in vitro*. ^∗^*P* < 0.05, ^∗∗^*P* < 0.01, statistically significant difference; error bars indicate ±SEM.

We next examined the migration and invasion abilities of C6, C6-A_1-3_, C6-A_2-3_, and C6-A_3-3_ cells using the scratch migration assay and transwell assay, respectively. As shown in Figure 3(a), there was a significant increase in the migration of C6-A_1-3_, C6-A_2-3_, and C6-A_3-3_ cells compared with that in the C6 control groups. Similarly, BM-MSC-conditioned medium treatment also enhanced glioma cell invasiveness (Figure 3(b)).

### 3.3. BM-MSC-Conditioned Medium Inhibits Growth but Promotes the Invasion of Glioma *In Vivo*

**Figure 4:**
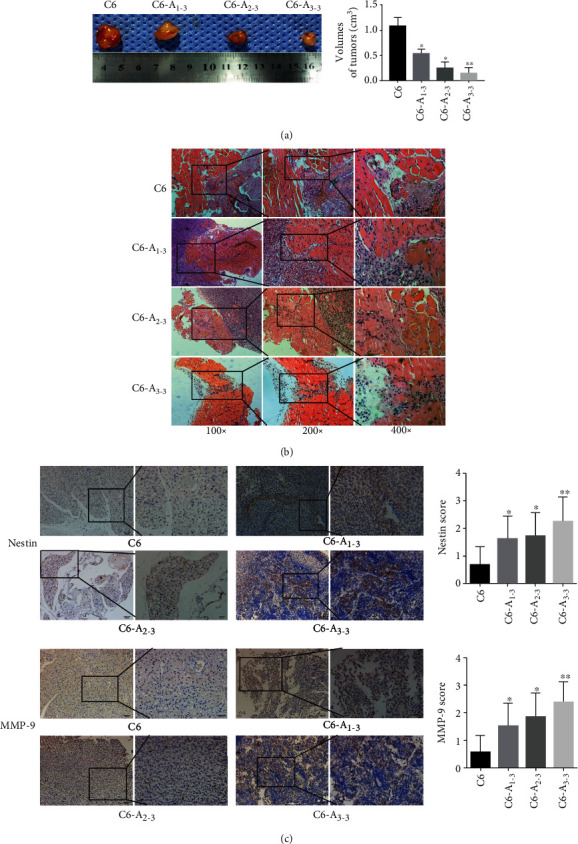
The influence of BM-MSC medium on the growth and invasion of xenografts in subcutaneously injected mice. (a) The volume of subcutaneous xenografts was smaller following BM-MSC-conditioned medium treatment, ^∗∗^*P* < 0.01. (b) H&E staining from the BM-MSC-conditioned medium treatment group showed an increased invasive tendency than that of the C6 group. Furthermore, the A_3-3_ group showed the highest invasiveness (100x, 200x, and 400x magnification). (c) IHC also indicated that the expression of nestin and MMP-9 were increased by BM-MSC-conditioned medium treatment, ^∗^*P* < 0.05, ^∗∗^*P* < 0.01. Original magnification 400x.

To determine the effect of BM-MSCs on transplanted glioma cells *in vivo*, C6, C6-A_1-3_, C6-A_2-3_, and C6-A_3-3_ cells were implanted into nude mice. After 14 days, tumor samples were collected. The tumor volumes in mice injected with BM-MSC-conditioned medium-treated tumor cells were significantly smaller than those in the control animals injected with C6 (Figure 4(a)). Hematoxylin and eosin (H&E) staining showed that the invasiveness of tumors in mice injected with the BM-MSC-conditioned medium-treated tumor cells increased significantly with prolonged treatment time (*P* < 0.05). Moreover, the invasiveness of C6-A_3-3_ was the strongest and that of the untreated C6 group was the weakest (Figure 4(b)). At the same time, IHC results also indicated that the expression of nestin and MMP-9 was increased in C6-A_3-3_ compared with the C6, C6-A_1-3_, and C6-A_2-3_ groups.

### 3.4. Identification of Differentially Expressed Proteins in C6 and C6 Treated with BM-MSC-Conditioned Medium

**Figure 5:**
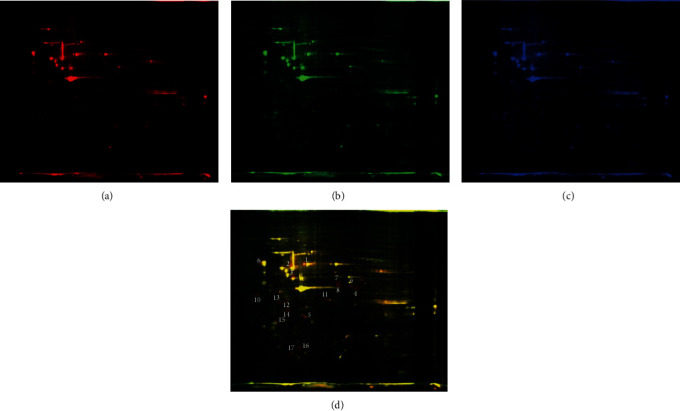
The result of 2D-DIGE shows 17 proteins differentially expressed between C6-A_3-3_ and C6 cells. (a) Cy3-labeled C6-A_3-3_. (b) Cy5-labeled C6. (c) Cy2 dye staining of the internal standard. (d) The merging of Cy3, Cy5, and Cy2 dye staining. The distribution of 17 differentially expressed protein spots in fluorescence difference gel electrophoresis gels. Protein spots are labeled (circle). The spots of interest were picked and subsequently identified by MALDI-TOF/TOF MS and peptide mass fingerprinting analysis, as reported in Table 1.

To better understand the differentially expressed proteins in C6 and C6 treated with the BM-MSC-conditioned medium, we analyzed the C6 control and C6-A_3-3_ using 2D-DIGE. Compared with the C6 control group, 17 proteins were differentially expressed in C6-A_3-3_. Among them, the expression of 5 protein spots was increased, and 12 protein spots were decreased in C6-A_3-3_ (Figure 5). These 17 protein spots were selected for identification using MALDI-TOF/TOF MS. The result showed that C6-A_3-3_ presented higher expression levels of vimentin (Vim), tubulin alpha-1C chain (Tuba1c), protein disulfide isomerase A6 (Pdia6), sphingosine kinase 1 (Sphk1), and annexin A4 (Anxa4), while C6 presented higher expression levels of calreticulin (Calr), ornithine aminotransferase (Oat), TAR DNA-binding protein 43 (Tardbp), beta-centractin (Actr1b), SET (Set), dimethylarginine dimethylaminohydrolase 1 (Ddah1), alpha-actinin-4 (Actn4), nucleophosmin (Npm1), thioredoxin-like protein 1 (Txnl1), tropomyosin beta chain (Tpm2), and probable ATP-dependent RNA helicase DDX28 (Ddx28) and ras-related protein Rap-2c (Rap2c) (Table 1). All the identified differential proteins were analyzed using PANTHER13.1 (http://pantherdb.org/). These proteins are potentially related to cell adhesion, differentiation, cell cytoskeleton and motility, and antioxidant function. They are widely distributed in the membrane, nucleus, cytoplasm, and mitochondria (see Supporting Information, Figure S1, and Table S1).

### 3.5. The Protein Expression Level Is Consistent with the Results of 2D-DIGE

**Figure 6:**
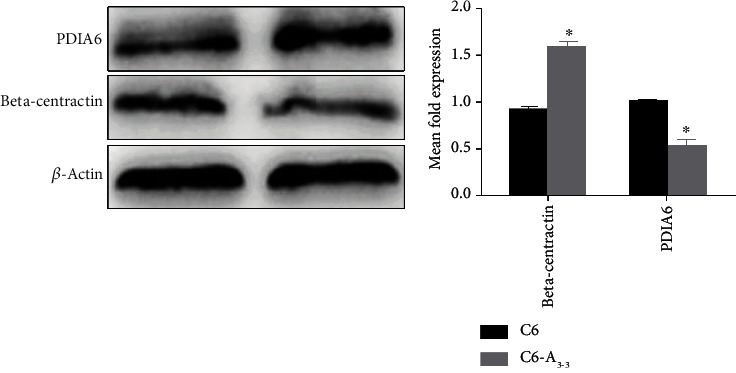
The level of protein expression of two identified differential proteins in C6-A_3-3_ and C6 cells. The expression level of the PDIA6 protein was higher in C6-A_3-3_ than C6 cells, but the expression level of beta-centractin protein was lower in C6-A_3-3_ than C6 cells, ^∗^*P* < 0.05. The change in protein expression was consistent with that of proteomic analysis.

Western blot confirmed one upregulated protein (PDIA6) and one downregulated protein (beta-centractin) in C6-A_3-3_ compared with the proteins in the C6 control group. Compared with the observations in the C6 control group, higher expression of PDIA6 and lower expression of beta-centractin were observed in C6-A_3-3_ cells. The results illustrated that the proteomic data based on 2D-DIGE were persuasive (Figure 6).

## 4. Discussion

Recently, stem cell-assisted gene therapy, including NSCs and BM-MSCs, has provided a promising treatment modality for various cancers. The robust tumor-tropic migratory capacities of stem cells can make them an excellent vector to deliver medicaments to the tumor [13–14, 16]. Compared with NSCs, BM-MSCs are easier to obtain and expand *in vitro*, and they do not have the limitation of potential immunologic incompatibility due to autologous transplantation. Therefore, BM-MSCs are more suitable for clinical applications than NSCs and have gained wide attention.

However, the effects of BM-MSCs on tumors are controversial, and the underlying mechanisms remain unknown. In this study, we provided evidence that the proliferation of glioma cells treated with the BM-MSC-conditioned medium was inhibited significantly both *in vitro* and *in vivo*, which is consistent with the results of several previous studies [17, 18]. However, some reports have stated that BM-MSCs contribute to the maintenance and progression of cancers, including glioma [19–21]. Our data also suggested that the migration and invasion of glioma cells treated with the BM-MSC-conditioned medium were promoted both *in vitro* and *in vivo*. Therefore, we should be more cautious when BM-MSCs are used as tumor-selective targeting carriers to deliver therapeutic agents to the tumor due to their potential cancer-promoting risk. BM-MSCs need to be genetically modified to express tumor-suppressive factors specifically. The integrated effects of MSCs on tumor cells depend on factors such as the host's immune status, different types of tumor models or sources of BM-MSCs, the microenvironment, and other unknown factors, accounting for the different results of protumorigenic or antitumorigenic effects *in vitro* and *in vivo*.

Furthermore, we compared the proteomic profile of BM-MSC-conditioned medium-treated C6-A_3-3_ cells with that of the untreated C6 cells using 2D-DIGE to elucidate the mechanism of the effect of BM-MSCs on C6. We identified 17 proteins differentially expressed in BM-MSC-treated C6-A_3-3_ with untreated C6 cells, which probably have a relationship with cell proliferation, metabolism, differentiation, antioxidation, the cell cytoskeleton, and motility. We screened 14 differentially expressed key candidate proteins that may be mainly related to cell proliferation, migration, and invasion and may contribute to the biological differences between C6-A_3-3_ and C6 cells.

In this report, six differentially expressed proteins were closely related to cell proliferation and differentiation, including Calr, SET, nucleophosmin, ornithine aminotransferase, dimethylarginine dimethylaminohydrolase 1, and TAR DNA-binding protein 43. Calr, a 46 kDa multifunctional protein, primarily maintains calcium homeostasis and is implicated in cell proliferation [22]. In recent studies, Calr overexpression has been linked to protumorigenic events in various cancers via regulation of the cell cycle or cancer cell angiogenesis [23, 24].

SET, also known as template activating factor-I *β* (TAF-I *β*), is a multifunctional oncoprotein that interacts with other proteins for regulating cellular signaling involved in the cell cycle and apoptosis [25]. SET has been reported to interact with p21^Cip1^ to modulate the cyclin E-CDK2 complex activity necessary for the G1/S transition and regulate cell division [26]. Moreover, SET can also directly bind to and inhibit cyclin E-CDK2, which is essential for mitotic onset [27].

Nucleophosmin (NPM1) is a phosphoprotein involved in maintaining genome stability and DNA repair proteins and regulating apoptosis [28, 29]. The expression of NPM1 is related to the mitotic index; moreover, the overexpression of NPM1 represents a poor prognosis for glioma patients [30]. NPM1 overexpression can increase ribosome biogenesis and protein synthesis and can accelerate DNA repair of tumor cells.

Oat, which is involved in glutamine metabolism, has been shown to regulate mitotic tumor cell division and promote cancer proliferation [31–33]. Moreover, the Oat gene is a target gene of *β*-catenin that is highly expressed in many cancers, including glioma [34]. Aberrant activation of the Wnt/*β*-catenin pathway leads to the dysregulation of target genes, such as Oat, to promote cancer progression [35].

Dimethylarginine dimethylaminohydrolase 1 (Ddah1) is a cysteine hydrolase enzyme responsible for the metabolism of asymmetric dimethylarginine, an endogenous inhibitor of nitric oxide synthase (iNOS). Recent reports have demonstrated that overexpression of Ddah1 enhances the expression of NO and vascular endothelial growth factor (VEGF) to promote angiogenesis and the growth of glioma *in vitro* and *in vivo* [36, 37]. Furthermore, upregulation of Ddah1 could induce the overexpression of target genes of NO, including VEGF, hypoxia-inducible factor 1-alpha (HIF-1*α*), c-Myc, and iNOS. These proteins are involved in various cellular energetic metabolic processes for cell proliferation [38].

TAR DNA-binding protein 43 (TDP-43) is a splicing factor belonging to the hnRNP family and plays an essential role in the RNA maturation process. It also participates in the regulation of the cell cycle and glucose or lipid metabolism [39]. Zeng et al. have also reported that TDP-43 increases melanoma proliferation by modulating glucose metabolism [40]. Moreover, TDP-43 can form a complex and interact with SRSF3 to regulate downstream genes, including PAR3 and NUMB, and then promote the proliferation and malignancy of mammary epithelial cells [41].

Furthermore, a group of differential proteins including protein disulfide isomerase A6 (PDIA6), sphingosine kinase 1 (Sphk1), Anx4a, vimentin, tubulin alpha-1C chain, beta-centractin, alph-actinin-4, ras-related protein Rap-2c, and tropomyosin beta chain showed a close relationship with motility and the cytoskeleton, which may modulate the invasion and migration of tumor cells.

PDIA6, belonging to the PDI family, has been shown to be involved in the disulfide exchange required for integrin-oriented adhesion [42]. Goplen et al. reported that PDI plays a vital role in the migration and invasion of gliomas [43]. The oncogenic cytokines derived from BM-MSCs may upregulate the expression of PDIA6, which reacts with the integrins in microenvironmental niches to facilitate cell-extracellular matrix adhesion, such as integrin *α*_2_*β*_1_, to promote the invasion and migration of glioma.

Sphk1 is a lipid kinase that induces the formation of sphingosine-1-phosphate (S1P) to interact with specific intracellular targets [44]. Novel evidence indicates that Sphk1/S1P may promote cancer cell transformation, epithelial-mesenchymal transition, and invasiveness [45, 46].

ANXA4 can interact with calcium ions and phospholipids to regulate vesicle aggregation, membrane repair, and synaptic exocytosis [47–49]. Knockdown of ANXA4 attenuates migration in breast cancer cells by regulating adhesion-related molecules [50]. Furthermore, the overexpression of ANXA4 has been identified in various malignant tumors, including glioma [51]. Our results are similar to those of a previous study, indicating that ANXA4 may be a vital characteristic in the growth of glioma cells under the stimulation of BM-MSCs.

Vimentin, a member of the type III IF protein family, is a canonical mesenchymal marker of epithelial-mesenchymal transition (EMT) characterized by the loss of cell adhesion and the acquisition of mesenchymal features [52, 53]. EMT has been identified as a key regulator of migration and invasion in some epithelial cancers [54, 55]. Several reports have demonstrated that the EMT process can be triggered, accompanied by the overexpression of vimentin and low expression of E-cadherin, and is involved in glioma cell migration and invasion [56].

Tubulin alpha-1C is an essential component of microtubules, which plays a vital role in axonal transportation in the nervous system [57]. Previous studies reported that the stable microtubule status plays an essential role in the process of tumor invasion [58]. As mentioned above, expression of vimentin was also increased in C6 with indirect stimulation of BM-MSCs. A recent study showed that vimentin filaments could promote the extension of tubulin-based microtentacles, suggesting that a possible mechanism to facilitate cancer invasion was provided by the coordination of vimentin and microtubules [59].

Beta-centractin plays a vital role in the formation and stabilization of immunological synapses and compromises the cytoskeletal superstructures at the postsynaptic size of neurons [60, 61]. Downregulation of beta-centractin might be involved in the dysfunction of dendritic cells and has been negatively correlated with the invasiveness of hepatocellular carcinoma [62].

Alph-actinin-4 (ACTN4), an actin-binding protein, modulates actin filament flexibility to regulate migration, invasion, and metastasis of cancer cells [63, 64]. Furthermore, studies from other groups have shown that the ACTN4-Akt axis promotes the degradation of GSK-3*β*, leading to the stabilization of *β*-catenin and enhancement of migration and invasion of cervical cancer cells [65].

Ras-related protein Rap-2c (Rap2c) is a member of the Ras superfamily of small GTPases [66]. Rap2c may function as a positive regulator of sterol regulatory element- (SRE-) mediated transcriptional regulation, which participates in the control of cell proliferation, differentiation, and migration [67]. A previous study demonstrated that the upregulation of Rap2c promoted the invasive and migratory capacities of osteosarcoma U2OS cells mediated by increased matrix metallopeptidase 2 (MMP-2) secretion and the Akt signaling pathway but had no effect on the proliferation or rate of apoptosis [68]. However, some reports have shown that Rap2c can suppress the EMT process of colorectal cancer and inhibit cancer cell migration and metastasis in a nuclear factor *κ*B- (NF-*κ*B-) dependent pathway [69]. Thus, Rap2c may play different roles in different cancers.

Tropomyosin beta chain (Tpm2) is essential for regulating muscle contraction by interacting with a complex of actin and troponin [70]. Moreover, it is also significant for various cell processes, including actin thin filament stabilization and cell migration. Previous research has shown that the overexpression of Tpm2 suppresses cell proliferation and migration by regulating RhoA signaling in colorectal cancer [71]. In addition, low expression of Tpm2 was also associated with an unfavorable prognosis in prostate and esophageal cancer patients [72, 73].

## 5. Conclusions

In conclusion, we revealed that the migration and invasion of glioma cells were promoted by BM-MSC-conditioned medium. However, the proliferation of tumor cells was inhibited significantly both *in vitro* and *in vivo*. Our data showed that some cytokines secreted from BM-MSCs might play a vital role in regulating oncoproteins or antioncoproteins responsible for the biological differences between BM-MSC-conditioned medium-treated C6 and untreated C6. The differentially expressed proteins are mostly involved in regulating cell proliferation, differentiation, cell cytoskeleton and motility, and metabolic and antioxidative functions. Therefore, we should be more cautious when BM-MSCs are used as tumor-selective targeting carriers to deliver therapeutic agents to the tumor. We need to focus on modified proteins that regulate the motility of glioma cells. Furthermore, the BM-MSCs need to be genetically modified to express tumor-suppressive factors specifically.

## Figures and Tables

**Table 1:** Upregulated protein expression and downregulated protein expression in C6-A_3-3_ compared with C6 cells.

Spot	Accession no.	Entry name	Protein name	%Cov	PI	MW (kDa)	FC	Protein score	*t* test, *P* value
*Increased protein expression*									
1	P31000	Vim	Vimentin	36	5.06	53.76	1.62	79	0.0017
2	Q6AYZ1	Tuba1c	Tubulin alpha-1C chain	27	4.96	50.59	1.52	63	0.0035
3	Q63081	Pdia6	Protein disulfide isomerase A6	41	4.95	48.49	1.71	175	0.0218
4	Q91V26	Sphk1	Sphingosine kinase 1	25	6.64	42.89	1.79	132	0.0183
5	P55260	Anxa4	Annexin A4	38	5.31	36.17	1.58	85	0.0415
*Decreased protein expression*									
6	P18418	Calr	Calreticulin	21	4.33	48.14	-1.51	103	0.0021
7	P04182	Oat	Ornithine aminotransferase	48	6.57	48.85	-1.75	147	0.0478
8	I6L9G6	Tardbp	TAR DNA-binding protein 43	29	5.85	45.05	-1.75	98	0.0196
9	B2RYJ7	Actr1b	Beta-centractin	25	5.98	42.38	-1.71	81	0.0361
10	Q63945	Set	SET	33	4.23	33.47	-1.87	161	0.0347
11	O08557	Ddah1	Dimethylarginine dimethylaminohydrolase 1	45	5.53	31.44	-1.67	127	0.0078
12	Q9QXQ0	Actn4	Alph-actinin-4	24	5.27	105.24	-1.62	168	0.0029
13	P13084	Npm1	Nucleophosmin	31	4.46	32.73	-1.96	119	0.0291
14	Q920J4	Txnl1	Thioredoxin-like protein 1	46	4.84	32.63	-1.52	91	0.0085
15	P58775	Tpm2	Tropomyosin beta chain	37	4.66	32.94	-1.62	182	0.0140
16	D4A7Q5	Ddx28	Probable ATP-dependent RNA helicase DDX28	43	10.43	59.74	-1.51	73	0.0382
17	D3ZK56	Rap2c	Ras-related protein Rap-2c	37	4.87	20.96	-2.34	153	0.0409

FC: fold change = C6-A_3-3_/C6.

## Data Availability

The datasets used and/or analyzed during the current study are available from the corresponding author on reasonable request.

## Abbreviations

ACN: Acetonitrile
Actn4: Alpha-actinin-4
Actr1b: Beta-centractin
Anxa4: Annexin A4
BM-MSCs: Bone marrow-derived mesenchymal stem cells
Calr: Calreticulin
CCK-8: Cell counting kit-8
CRT: Calreticulin
Ddah1: Dimethylarginine dimethylaminohydrolase 1
2-D DIGE: Two-dimensional fluorescence difference gel electrophoresis
Ddx28: Probable ATP-dependent RNA helicase DDX28
DMEM: Dulbecco's modified Eagle's medium
EMT: Epithelial mesenchymal transition
FBS: Fetal bovine serum
GBM: Glioblastoma multiforme
H&E: Hematoxylin and eosin
IPG: Immobilized pH gradient
MALDI-TOF/TOF MS: Matrix-assisted laser desorption ionization time-of-flight mass spectrometry
Npm1: Nucleophosmin
Oat: Ornithine aminotransferase
Pdia6: Protein disulfide-isomerase A6
Rap2c: Ras-related protein Rap-2c
SDS: Sodium dodecyl sulfate
SET: Set
S1P: Sphingosine-1-phosphate
S1P1-5: Five S1P-specific G protein coupled receptors
Sphk1: Sphingosine kinase 1
TAF-I *β*: Template activating factor-I *β*
Tardbp: TAR DNA-binding protein 43
TFA: Trifluoroacetic acid
Tpm2: Tropomyosin beta chain
Tuba1c: Tubulin alpha-1C chain
Txnl1: Thioredoxin-like protein 1
Vim: Vimentin.

## Supplementary Materials

Supplementary Materials Including the expression profile and functional annotation of the significantly differentiated expressed proteins (Figure S1, Table S1) and a diagrammatic drawing of coculture (Figure S2).

## References

[1] Ostrom Q. T., Bauchet L., Davis F. G. (2014). The epidemiology of glioma in adults: a "state of the science" review. *Neuro-Oncology*.

[2] Omuro A., DeAngelis L. M. (2013). Glioblastoma and other malignant gliomas: a clinical review. *JAMA*.

[3] Tobias A., Ahmed A., Moon K. S., Lesniak M. S. (2013). The art of gene therapy for glioma: a review of the challenging road to the bedside. *Journal of Neurology, Neurosurgery, and Psychiatry*.

[4] Shinojima N., Hossain A., Takezaki T. (2013). TGF-*β* mediates homing of bone marrow-derived human mesenchymal stem cells to glioma stem cells. *Cancer Research*.

[5] Nakamizo A., Marini F., Amano T. (2005). Human bone marrow-derived mesenchymal stem cells in the treatment of gliomas. *Cancer Research*.

[6] Studeny M., Marini F. C., Champlin R. E., Zompetta C., Fidler I. J., Andreeff M. (2002). Bone marrow-derived mesenchymal stem cells as vehicles for interferon-beta delivery into tumors. *Cancer Research*.

[7] Wang C., Meng H., Wang X., Zhao C., Peng J., Wang Y. (2016). Differentiation of bone marrow mesenchymal stem cells in osteoblasts and adipocytes and its role in treatment of osteoporosis. *Medical science monitor : international medical journal of experimental and clinical research*.

[8] Otsu K., Das S., Houser S. D., Quadri S. K., Bhattacharya S., Bhattacharya J. (2009). Concentration-dependent inhibition of angiogenesis by mesenchymal stem cells. *Blood*.

[9] Clarke M. R., Imhoff F. M., Baird S. K. (2015). Mesenchymal stem cells inhibit breast cancer cell migration and invasion through secretion of tissue inhibitor of metalloproteinase-1 and -2. *Molecular Carcinogenesis*.

[10] Suzuki K., Sun R., Origuchi M. (2011). Mesenchymal stromal cells promote tumor growth through the enhancement of neovascularization. *Molecular Medicine*.

[11] Shou K., Niu Y., Zheng X. (2017). Enhancement of bone-marrow-derived mesenchymal stem cell angiogenic capacity by NPWT for a combinatorial therapy to promote wound healing with large defect. *BioMed Research International*.

[12] Liu Z., Li L., Yang Z. (2010). Increased expression of MMP9 is correlated with poor prognosis of nasopharyngeal carcinoma. *BMC cancer*.

[13] Aboody K. S., Brown A., Rainov N. G. (2000). Neural stem cells display extensive tropism for pathology in adult brain: evidence from intracranial gliomas. *Proceedings of the National Academy of Sciences of the United States of America*.

[14] Lee D. H., Ahn Y., Kim S. U. (2009). Targeting rat brainstem glioma using human neural stem cells and human mesenchymal stem cells. *Clinical cancer research : an official journal of the American Association for Cancer Research*.

[15] Li C., Wei G., Gu Q. (2015). Donor age and cell passage affect osteogenic ability of rat bone marrow mesenchymal stem cells. *Cell Biochemistry and Biophysics*.

[16] Menon L. G., Kelly K., Yang H. W., Kim S. K., Black P. M., Carroll R. S. (2009). Human bone marrow-derived mesenchymal stromal cells expressing S-TRAIL as a cellular delivery vehicle for human glioma therapy. *Stem cells*.

[17] Ho I. A., Toh H. C., Ng W. H. (2013). Human bone marrow-derived mesenchymal stem cells suppress human glioma growth through inhibition of angiogenesis. *Stem cells*.

[18] Kolosa K., Motaln H., Herold-Mende C., Korsic M., Lah T. T. (2015). Paracrine effects of mesenchymal stem cells induce senescence and differentiation of glioblastoma stem-like cells. *Cell Transplantation*.

[19] Abdi Z., Eskandary H., Nematollahi-Mahani S. N. (2018). Effects of two types of human cells on outgrowth of human glioma in rats. *Turkish Neurosurgery*.

[20] Zhang T., Lee Y. W., Rui Y. F., Cheng T. Y., Jiang X. H., Li G. (2013). Bone marrow-derived mesenchymal stem cells promote growth and angiogenesis of breast and prostate tumors. *Stem Cell Research & Therapy*.

[21] Liu J., Zhang Y., Bai L., Cui X., Zhu J. (2012). Rat bone marrow mesenchymal stem cells undergo malignant transformation via indirect co-cultured with tumour cells. *Cell Biochemistry and Function*.

[22] Zamanian M., Veerakumarasivam A., Abdullah S., Rosli R. (2013). Calreticulin and cancer. *Pathology oncology research : POR*.

[23] Chiang W. F., Hwang T. Z., Hour T. C. (2013). Calreticulin, an endoplasmic reticulum-resident protein, is highly expressed and essential for cell proliferation and migration in oral squamous cell carcinoma. *Oral Oncology*.

[24] Chen C. N., Chang C. C., Su T. E. (2009). Identification of calreticulin as a prognosis marker and angiogenic regulator in human gastric cancer. *Annals of Surgical Oncology*.

[25] Bayarkhangai B., Noureldin S., Yu L. (2018). A comprehensive and perspective view of oncoprotein SET in cancer. *Cancer Medicine*.

[26] Estanyol J. M., Jaumot M., Casanovas O., Rodriguez-Vilarrupla A., Agell N., Bachs O. (1999). The protein SET regulates the inhibitory effect of p21^Cip1^ on cyclin E-cyclin-dependent kinase 2 activity∗. *The Journal of Biological Chemistry*.

[27] Canela N., Rodriguez-Vilarrupla A., Estanyol J. M. (2003). The SET protein regulates G_2_/M transition by modulating cyclin B-cyclin-dependent kinase 1 activity∗. *The Journal of Biological Chemistry*.

[28] Grisendi S., Mecucci C., Falini B., Pandolfi P. P. (2006). Nucleophosmin and cancer. *Nature Reviews Cancer*.

[29] Di Matteo A., Franceschini M., Chiarella S., Rocchio S., Travaglini-Allocatelli C., Federici L. (2016). Molecules that target nucleophosmin for cancer treatment: an update. *Oncotarget*.

[30] Holmberg Olausson K., Elsir T., Moazemi Goudarzi K., Nister M., Lindstrom M. S. (2015). NPM1 histone chaperone is upregulated in glioblastoma to promote cell survival and maintain nucleolar shape. *Scientific Reports*.

[31] Brosnan M. E., Brosnan J. T. (2009). Hepatic glutamate metabolism: a tale of 2 hepatocytes. *The American journal of clinical nutrition*.

[32] Wang G., Shang L., Burgett A. W., Harran P. G., Wang X. (2007). Diazonamide toxins reveal an unexpected function for ornithine -amino transferase in mitotic cell division. *Proceedings of the National Academy of Sciences of the United States of America*.

[33] Miyasaka Y., Enomoto N., Nagayama K. (2001). Analysis of differentially expressed genes in human hepatocellular carcinoma using suppression subtractive hybridization. *British Journal of Cancer*.

[34] Zigmond E., Ben Ya'acov A., Lee H. (2015). Suppression of hepatocellular carcinoma by inhibition of overexpressed ornithine aminotransferase. *ACS Medicinal Chemistry Letters*.

[35] Nejak-Bowen K. N., Monga S. P. (2011). Beta-catenin signaling, liver regeneration and hepatocellular cancer: sorting the good from the bad. *Seminars in Cancer Biology*.

[36] Kostourou V., Robinson S. P., Cartwright J. E., Whitley G. S. (2002). Dimethylarginine dimethylaminohydrolase I enhances tumour growth and angiogenesis. *British Journal of Cancer*.

[37] Smith C. L., Birdsey G. M., Anthony S., Arrigoni F. I., Leiper J. M., Vallance P. (2003). Dimethylarginine dimethylaminohydrolase activity modulates ADMA levels, VEGF expression, and cell phenotype. *Biochemical and Biophysical Research Communications*.

[38] Chang C. F., Diers A. R., Hogg N. (2015). Cancer cell metabolism and the modulating effects of nitric oxide. *Free Radical Biology & Medicine*.

[39] Park Y. Y., Kim S. B., Han H. D. (2013). Tat-activating regulatory DNA-binding protein regulates glycolysis in hepatocellular carcinoma by regulating the platelet isoform of phosphofructokinase through microRNA 520. *Hepatology*.

[40] Zeng Q., Cao K., Liu R. (2017). Identification of TDP-43 as an oncogene in melanoma and its function during melanoma pathogenesis. *Cancer Biology & Therapy*.

[41] Ke H., Zhao L., Zhang H. (2018). Loss of TDP43 inhibits progression of triple-negative breast cancer in coordination with SRSF3. *Proceedings of the National Academy of Sciences of the United States of America*.

[42] Lahav J., Wijnen E. M., Hess O. (2003). Enzymatically catalyzed disulfide exchange is required for platelet adhesion to collagen via integrin alpha2beta1. *Blood*.

[43] Goplen D., Wang J., Enger P. O. (2006). Protein disulfide isomerase expression is related to the invasive properties of malignant glioma. *Cancer Research*.

[44] Pyne N. J., Pyne S. (2010). Sphingosine 1-phosphate and cancer. *Nature Reviews Cancer*.

[45] Pyne S., Adams D. R., Pyne N. J. (2016). Sphingosine 1-phosphate and sphingosine kinases in health and disease: recent advances. *Progress in Lipid Research*.

[46] Ogretmen B. (2018). Sphingolipid metabolism in cancer signalling and therapy. *Nature Reviews Cancer*.

[47] Yao H., Sun C., Hu Z., Wang W. (2016). The role of annexin A4 in cancer. *Frontiers in bioscience*.

[48] Willshaw A., Grant K., Yan J. (2004). Identification of a novel protein complex containing annexin A4, rabphilin and synaptotagmin. *FEBS Letters*.

[49] Kaetzel M. A., Mo Y. D., Mealy T. R. (2001). Phosphorylation mutants elucidate the mechanism of annexin IV-mediated membrane aggregation. *Biochemistry*.

[50] Zimmermann U., Balabanov S., Giebel J. (2004). Increased expression and altered location of annexin IV in renal clear cell carcinoma: a possible role in tumour dissemination. *Cancer Letters*.

[51] Wei B., Guo C., Liu S., Sun M. Z. (2015). Annexin A4 and cancer. *Clinica chimica acta; international journal of clinical chemistry*.

[52] Satelli A., Li S. (2011). Vimentin in cancer and its potential as a molecular target for cancer therapy. *Cellular and molecular life sciences : CMLS*.

[53] Iser I. C., Pereira M. B., Lenz G., Wink M. R. (2017). The epithelial-to-mesenchymal transition-like process in glioblastoma: an updated systematic review and in silico investigation. *Medicinal Research Reviews*.

[54] Moustakas A., Heldin P. (2014). TGF*β* and matrix-regulated epithelial to mesenchymal transition. *Biochimica et Biophysica Acta*.

[55] Lamouille S., Xu J., Derynck R. (2014). Molecular mechanisms of epithelial-mesenchymal transition. *Nature Reviews Molecular Cell Biology*.

[56] Liu C. A., Chang C. Y., Hsueh K. W. (2018). Migration/invasion of malignant gliomas and implications for therapeutic treatment. *International Journal of Molecular Sciences*.

[57] Wade R. H. (2009). On and around microtubules: an overview. *Molecular Biotechnology*.

[58] Korb T., Schluter K., Enns A. (2004). Integrity of actin fibers and microtubules influences metastatic tumor cell adhesion. *Experimental Cell Research*.

[59] Whipple R. A., Balzer E. M., Cho E. H., Matrone M. A., Yoon J. R., Martin S. S. (2008). Vimentin filaments support extension of tubulin-based microtentacles in detached breast tumor cells. *Cancer Research*.

[60] Andrews D. M., Andoniou C. E., Scalzo A. A. (2005). Cross-talk between dendritic cells and natural killer cells in viral infection. *Molecular Immunology*.

[61] Cuadrado-Tejedor M., Sesma M. T., Gimenez-Amaya J. M., Ortiz L. (2005). Changes in cytoskeletal gene expression linked to MPTP-treatment in mice. *Neurobiology of Disease*.

[62] Weng Y. Q., Qiu S. J., Liu Y. K., Fan J., Gao Q., Tang Z. Y. (2008). Down-regulation of beta-centractin might be involved in dendritic cells dysfunction and subsequent hepatocellular carcinoma immune escape: a proteomic study. *Journal of Cancer Research and Clinical Oncology*.

[63] Kumeta M., Yoshimura S. H., Harata M., Takeyasu K. (2010). Molecular mechanisms underlying nucleocytoplasmic shuttling of actinin-4. *Journal of Cell Science*.

[64] Shao H., Wang J. H., Pollak M. R. (2010). *α*-Actinin-4 is essential for maintaining the spreading, motility and contractility of fibroblasts. *PLoS One*.

[65] An H. T., Yoo S. (2016). *α*-Actinin-4 induces the epithelial-to-mesenchymal transition and tumorigenesis via regulation of Snail expression and *β*-catenin stabilization in cervical cancer. *Oncogene*.

[66] Pasheva E., Janoueix-Lerosey I., Tavitian A., de Gunzburg J. (1994). Characterization of the Ras-related RAP2A protein expressed in the baculovirus-insect cell system: processing of the protein in insect cells and comparison with the bacterially produced unprocessed form. *Biochemical and Biophysical Research Communications*.

[67] Guo Z., Yuan J., Tang W. (2007). Cloning and characterization of the human gene RAP2C, a novel member of Ras family, which activates transcriptional activities of SRE. *Molecular Biology Reports*.

[68] Wu J., Du W., Wang X. (2018). Ras-related protein Rap2c promotes the migration and invasion of human osteosarcoma cells. *Oncology Letters*.

[69] Shen Z., Zhou R., Liu C. (2017). MicroRNA-105 is involved in TNF-*α*-related tumor microenvironment enhanced colorectal cancer progression. *Cell Death & Disease*.

[70] Tajsharghi H., Ohlsson M., Palm L., Oldfors A. (2012). Myopathies associated with *β*-tropomyosin mutations. *Neuromuscular disorders*.

[71] Cui J., Cai Y., Hu Y. (2016). Epigenetic silencing of TPM2 contributes to colorectal cancer progression upon RhoA activation. *Tumour biology : the journal of the International Society for Oncodevelopmental Biology and Medicine*.

[72] Jazii F. R., Najafi Z., Malekzadeh R. (2006). Identification of squamous cell carcinoma associated proteins by proteomics and loss of beta tropomyosin expression in esophageal cancer. *World Journal of Gastroenterology*.

[73] Varisli L. (2013). Identification of new genes downregulated in prostate cancer and investigation of their effects on prognosis. *Genetic Testing and Molecular Biomarkers*.

