# Supplementary figures and images for: Bone Marrow-Derived Mesenchymal Stem Cells Differentially Affect Glioblastoma Cell Proliferation, Migration, and Invasion: A 2D-DIGE Proteomic Analysis
Source: Biomed Res Int. 2021 Feb 11;2021:4952876. doi: 10.1155/2021/4952876 (PMC7892224; doi:10.1155/2021/4952876)

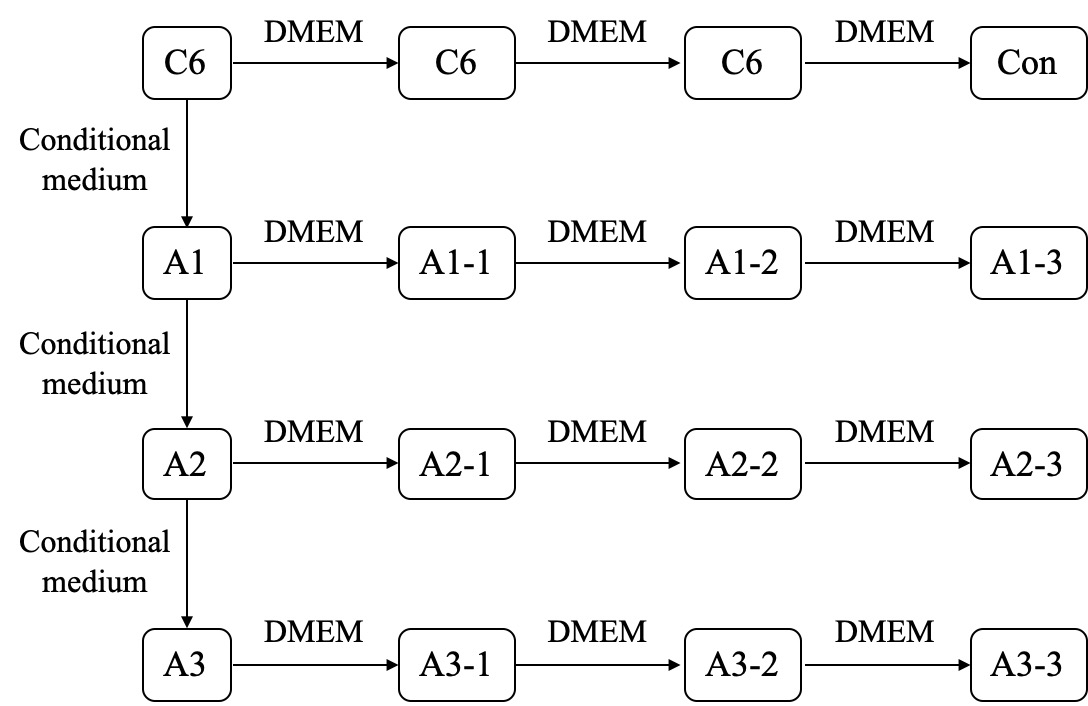


A diagrammatic drawing of co-culture

Supplement: Supplementary Materials — Including the expression profile and functional annotation of the significantly differentiated expressed proteins (Figure S1, Table S1) and a diagrammatic drawing of coculture (Figure S2). [file 4952876.f1.docx]
